# Adenovirus Gene Transfer to *Amelogenesis Imperfecta* Ameloblast-Like Cells

**DOI:** 10.1371/journal.pone.0024281

**Published:** 2011-10-07

**Authors:** Anton V. Borovjagin, Juan Dong, Michael J. Passineau, Changchun Ren, Ejvis Lamani, Olga A. Mamaeva, Hongju Wu, Enid Keyser, Miho Murakami, Shuo Chen, Mary MacDougall

**Affiliations:** 1 Department of Periodontics, University of Alabama at Birmingham School of Dentistry, Birmingham, Alabama, United States of America; 2 Department of Orthodontics, University of Alabama at Birmingham School of Dentistry, Birmingham, Alabama, United States of America; 3 Department of Oral and Maxillofacial Surgery, University of Alabama at Birmingham School of Dentistry, Birmingham, Alabama, United States of America; 4 Institute of Oral Health Research, University of Alabama at Birmingham School of Dentistry, Birmingham, Alabama, United States of America; 5 Department of Obstetrics and Gynecology, University of Alabama at Birmingham, Birmingham, Alabama, United States of America; 6 Division of Human Gene Therapy, Department of Medicine, The Gene Therapy Center, University of Alabama at Birmingham, Birmingham, Alabama, United States of America; 7 Division of Cardiovascular Medicine and Allegheny-Singer Research Institute, West-Penn Allegheny Health System, Pittsburgh, Pennsylvania, United States of America; 8 Department of Pediatric Dentistry, Dental School University of Texas Health Science Center at San Antonio, San Antonio, Texas, United States of America; University of Giessen Lung Center, Germany

## Abstract

To explore gene therapy strategies for amelogenesis imperfecta (AI), a human ameloblast-like cell population was established from third molars of an AI-affected patient. These cells were characterized by expression of cytokeratin 14, major enamel proteins and alkaline phosphatase staining. Suboptimal transduction of the ameloblast-like cells by an adenovirus type 5 (Ad5) vector was consistent with lower levels of the coxsackie-and-adenovirus receptor (CAR) on those cells relative to CAR-positive A549 cells. To overcome CAR -deficiency, we evaluated capsid-modified Ad5 vectors with various genetic capsid modifications including “pK7” and/or “RGD” motif-containing short peptides incorporated in the capsid protein fiber as well as fiber chimera with the Ad serotype 3 (Ad3) fiber “knob” domain. All fiber modifications provided an augmented transduction of AI-ameloblasts, revealed following vector dose normalization in A549 cells with a superior effect (up to 404-fold) of pK7/RGD double modification. This robust infectivity enhancement occurred through vector binding to both α_v_β3/α_v_β5 integrins and heparan sulfate proteoglycans (HSPGs) highly expressed by AI-ameloblasts as revealed by gene transfer blocking experiments. This work thus not only pioneers establishment of human AI ameloblast-like cell population as a model for *in vitro* studies but also reveals an optimal infectivity-enhancement strategy for a potential Ad5 vector-mediated gene therapy for AI.

## Introduction

Enamel is produced by highly specialized epithelial ameloblasts that differentiate from the inner enamel epithelium originating from the enamel organ epithelium (EOE) [1], [2]. Secretory ameloblasts synthesize and secrete a number of enamel proteins that include: the most abundant enamel matrix protein amelogenin (Amel) [3], ameloblastin (Ambn) [4], [5], the largest secreted enamel glycoprotein enamelin (Enam) [6], amelotin (Amtn) [7], and odontogenic ameloblast-associated protein (ODAM, also known as Apin) [8]. Enamel matrix also contains two major matrix proteinases: matrix metalloproteinase 20 (MMP-20, also known as enamelysine) [9] and kallekrein 4 (KLK 4; also known as EMSP 1) [10]–[12]. During the ameloblast secretory phase MMP-20 and KLK 4 augment matrix mineralization through proteolytic degradation of Amel and other matrix proteins. This leads to the removal of enamel's organic components, followed by apoptosis of ameloblasts [13].

Non-syndromic genetic diseases affecting enamel formation have been broadly classified as amelogenesis imperfecta (AI). The most prevalent autosomal dominant (AD) form of AI is commonly caused by the *ENAM* gene mutations, while the X-linked form of AI is caused by alterations in the *AMELX* gene [14]. Several treatment options have been described to rehabilitate AI patients, ranging from preventive intervention to interim composite restorations, orthodontic treatment, orthognathic surgery and placement of cast crowns [15].

The complex secretory pattern of ameloblasts and narrow developmental window for tooth formation makes this process highly susceptible to disruption resulting in irreparable tooth malformation early in an affected individual's life. The restricted temporal pattern of amelogenesis suggests feasibility of ameloblast-targeted gene replacement strategy aimed at rescuing the AI-associated phenotype by transiently replacing defective gene products in the ameloblast cellular layer within this time window. A gene delivery vector, which could be injected into ameloblasts to accomplish this aim, offers a conceptually plausible approach to restoring tooth formation in AI-afflicted individuals.

A gene therapy vector capable of efficient delivery and expression of genes in ameloblasts is pivotal for such a strategy. Human adenovirus type 5 (Ad5)-based vectors offer the strongest *in vivo* transgene expression of limited duration without host DNA integration [16], [17], thereby minimizing human safety concerns. Further, advanced-generation “gutless” Ad vectors with reduced immunogenic properties [18], [19] could potentially be employed to minimize immune response in patients and prolong expression of the therapeutic/replacement gene if necessary. Those factors make Ad an attractive system for localized time-limited gene expression in ameloblasts. Improving the efficiency of target cell transduction to minimize viral load and the associated Ad immune response is pivotal for clinical applications of the above gene therapy strategies.

Tissues vary in their susceptibility to infection by Ad5, based primarily upon their expression of the native Ad5 receptor, Coxsackie-and-Adenovirus Receptor (CAR) [20]. Susceptibility of ameloblasts to Ad5 infection has not been addressed to date due to the lack of a human AI-ameloblast culture as a model for exploring gene delivery strategies. Our purpose, therefore, was to establish a primary human AI ameloblast-like cell population and evaluate the utility of tropism-modified Ad5 vectors for effective delivery to these cells as a potential gene therapy strategy for AI. This study represents a first step towards optimization of gene delivery strategy for AI ameloblasts using Ad5-based vectors.

## Materials and Methods

### Cell Lines

Human alveolar basal epithelial adenocarcinoma A549 cells as well as rhabdomyosarcoma cell line (RD) were obtained from American Tissue Culture Collection (ATCC). All cells were cultured in 10% fetal bovine serum (FBS) (HyClone, Logan UT, USA) supplemented Dulbecco's Modified Eagle's Medium and Ham's F-12 Nutrient (50∶50) Mixture (DMEM/F-12) with 2 mM L-glutamine and 100 µg/ml Penicillin/Streptomycin (Mediatech, Herndon, VA USA).

### Establishment of Human AI Ameloblast-like Cells

Extracted third molars from a 14 year-old female patient presenting with a spontaneous case of AI (IRB approval from University of Texas Health Science Center at San Antonio) were obtained with written informed consent of the parent. Crown EOE tissue was dissected and placed in explant cultures as previously described [21]. An ameloblast primary cell population (AI-WAm) was cultured, expanded and frozen stocks prepared.

### Immunohistochemistry

Cells (passage 4–6) were grown in 4-well chamber slides, fixed with 4% formaldehyde (10 minutes at room temperature), and permeabilized with 0.25% Triton X-100. After washes in PBS, the samples were blocked with 10% BSA (Sigma, St. Louis, MO) and incubated overnight at 4°C with one of the following primary antibodies: rabbit polyclonal anti-Amel (Sigma, St. Louis, MO), rabbit polyclonal anti-cytokeratin 14 (ab53115; Abcam, Cambridge, MA); goat polyclonal anti-Enam (C-18; Santa Cruz Biotechnology, Santa Cruz, CA), mouse anti-human syndecan 4 (Abcam, Cambridge, MA) at 1∶50 dilutions in 3% BSA/PBS or mouse monoclonal anti-hCAR antibody (clone RmcB, Millipore, Billerica, MA), mouse monoclonal anti-human HSPG/GAG 10E4 antibody (F58-10E4, Seikagaku Biobusiness Corp), mouse anti-human integrin α_v_β3 (LM609 clone) or α_v_β5 (P1F6 clone) monoclonal antibodies (500 µg/ml) (Millipore, Billerica, MA) at 1∶100 dilutions in 3% BSA/PBS at room temperature for 2 hrs. The cells were washed with PBS followed by incubation with anti-rabbit or anti-goat HRP polymer conjugate (SuperPicTure™ Polymer Detection Kit) for Amel, Enam and cytokeratin 14 and the color reaction developed according to instructions. For CAR, HSPG or integrin staining, the cells were washed with PBS, incubated for an hour with Alexa Fluor 488-conjugated goat anti-mouse IgG (1∶1000, Invitrogen Molecular Probes, Carlsbad, CA), washed and counterstained at room temperature for 1 minute with 300 nM DAPI (Invitrogen Molecular Probes, Carlsbad, CA) for nuclear staining. All samples were mounted with Crystal/Mount (Biomeda, Foster City, CA) and visualized using a Nikon inverted microscope (Nikon Instruments Inc., Melville, NY). All images were taken with Roper Scientific digital camera using the same exposure time (300 ms for FITC and 30 ms for DAPI images) using either 10×, 40× or a 60× objectives and overlaid using the NIS-Element AR™ software.

### *In situ* Alkaline Phosphatase (ALP) Histochemistry

ALP *in situ* histochemistry of AI-WAm cells and mouse 3-day postnatal tooth sections were performed as described previously [22].

### Viral Vectors

All Ad5 vectors used in this study were replication-deficient (*E1*-deleted). Construction of the capsid-modified vectors: Ad5 (G/L), Ad5-RGD (G/L), Ad5-pK7 (G/L) and Ad5-pK7/RGD (G/L) containing two reporter genes: Green Fluorescent Protein (GFP) and firefly luciferase (Luc), each driven by a separate cytomegalovirus (CMV) promoter, was described previously [23]. The serotype chimera fiber modification was represented by two different vectors: Ad5/3 (L) [24] and Ad5/3 (G), constructed by homologous recombination in bacteria [25] using a shuttle vector pKAN.F5/3 [26] and a pVK900 backbone with deleted fiber gene and CMV-GFP cassette in place of the *E1* region (Krasnykh, unpublished plasmid).

### *In vitro* Gene Transfer Assay

Infectivity of the Ad vectors with native and modified fibers in AI-WAm cells was determined in gene transfer experiments using expression of two different reporters: GFP (G) and Luc (L). TCID_50_ titers for CsCl-purified viral preparations were determined by the Kärber equation [27] in HEK293 cells.

Transduction of all cells with unmodified vector Ad5 (G/L) was carried out at the infection dose of either 10 or 50 TCID_50_/cell, whereas doses of fiber-modified Ads retaining CAR-tropism or Ads re-targeted to Ad3 receptors (Ad5/3) were empirically adjusted in our preliminary experiments by Luc expression levels in CAR/CD46-positive A549 cells [28]. Besides, comparable levels of the reporter gene (Luc or GFP) expression from all the vectors in A549 cells were verified in parallel for every gene transfer experiment to control the accuracy of vector dosing. All luciferase assays were performed at 20 hours post infection using a luciferase assay kit (Promega, Madison, WI). Expression of GFP reporter was analyzed by GFP fluorescence of infected cells visualized using a Nikon inverted microscope with Nikon digital camera (Nikon Instruments Inc., Melville, NY) and quantified using multifunctional Synergy HT plate reader (Bio-Tek Instruments, Winooski, VT, USA). Bioluminescence was quantified using a luminometer (Femtomaster, Zylux, Germany).

The lack of replication competent adenovirus (RCA) contamination in vector preparations was verified for each vector by determining the *E4* copy number in infected AI-WAm cells at 6, 20 and 36 hrs post infection. Extraction of total DNA from Ad-infected cells was carried out using DNeasy Kit (Qiagen, Valencia, CA) according to the manufacturer's instructions. No statistically significant increase in *E4* copy number over time was considered as an evidence for the lack of RCA contamination in viral preparations that could potentially affect the results of our gene transfer assays via amplification of the transgene expression.

### Blocking experiments

A 1∶1 mixture of purified recombinant α_v_β3/α_v_β5 integrins (2 µg) or either 5 or 50 µg of heparin were used as competitors for cellular integrins and HSPGs, respectively. Various fiber-modified Ad5 vectors were pre-incubated with the competitors in 50 µl of 2% FBS medium for 30 minutes at room temperature prior to infection of 5×10^3^ AI-WAm cells in 96-well plate at an MOI of 10 TCID_50_/cell.

The gene transfer (Luc) assay was then performed as described above.

### RNA isolation and cDNA synthesis

Total RNA was isolated from cells using RNeasy Mini Kit (Qiagen, Valencia, CA) and treated with DNase1 (Qiagen) to eliminate DNA contamination. First-strand cDNA was synthesized using 1 µg of RNA and M-MLV reverse transcriptase kit (Applied Biosystems, Foster City, CA) according to the manufacturer's protocol.

### Quantification of mRNA by Real Time PCR

Quantitative real-time PCR (qPCR) was performed with a 7500 Applied Biosystems Real-Time PCR Detection System (Foster City, CA) using a SYBR Green Master mix (SABiosciences, Frederick, MD). Relative gene expression was presented as Δ*C*_T_ values calculated for each gene's cDNA by normalization to *C*_T_ values for glyceraldehyde-3-phosphate dehydrogenase (*GAPDH*) as a housekeeping gene or as “fold difference” calculated for each mRNA level in a given cell type relative to that in RD (control) cells using the “2^−ΔΔ*C*T^ method” [29]. Primers for *AMELX*, *ENAM*, *AMBN*, *AMTN* and *ODAM* genes were purchased from SABiosciences. Primers used for quantification of *hCAR* by qPCR were described previously [30]. The following PCR primers were used for quantification of *hCD46*: Forward: 5′-CTTTCCTTCCTGGCGCTTTC-3′; Reverse: 5′-CGGAGAAGGAGTACAGCAGCA-3′ and *GAPDH*: Forward: 5′-AGGTCGGTGTGAACGGATTTG-3′; Reverse: 5′-TGTAGACCATGTAGTTGAGGTCA-3′.

### Quantification of Adenoviral genomes

DNA from infected AI-WAm cells was isolated using a DNeasy Kit (Qiagen, Valencia, CA). Ad5 *E4* gene copy number in each sample was determined by q-PCR using a Taqman *E4* probe (5′–FAM-TGGCATGACACTACGACCAACAC GATCT-TAMRA-3′) and *E4*-specific PCR primers (Forward: 5′-GAGTGCGCCGAGACAAC-3′; Reverse: 5′-TGGATGCCACAGGATTCCAT-3′). Human β-actin primers were as described previously [31]. *E4* copy number values were calculated from sample C_T_ values based on a standard curve obtained by serial dilutions of purified Ad5 genomic DNA with known *E4* copy number as described previously [31], [32]. QPCR of the β-actin gene in the same samples was performed to quantify total cellular DNA using a standard curve generated from serial dilutions of known amount of total cellular DNA isolated from AI-WAm cells. The *E4* copy number values (measured in triplicate) were normalized for total AI-WAm cellular DNA in each sample.

### Flow Cytometry

Expression of CAR, α_v_β3/α_v_β5 integrins, heparan sulfate proteoglycans (HSPG), syndecan 4, or CD46 was analyzed by flow cytometry (FACS) method using a Becton-Dickinson FACSCalibur device (UAB FACS Core). Confluent cells were collected by digestion with Versene, pelleted at 1000 rpm and resuspended in 0.5 ml of 5% FBS/PBS. Cells (2×10^5^/sample) were incubated at 4°C for 1 hr with one of the following antibodies: human CAR-specific monoclonal antibody RmcB (Millipore, Billerica, MA) at 1∶100 dilution, anti-α_v_β3 integrin (LM609 clone) or anti-α_v_β5 integrin (P1F6 clone) monoclonal antibody (Millipore, Billerica, MA), each at 5 µg/ml final concentration, anti-syndecan 4 antibody (Abcam, Cambridge, MA) at 1∶50 dilution, anti-HSPG 10E4 antibody (F58-10E4, Seikagaku Biobusiness Corp) at 1∶100 dilution or anti-human CD46/isotype control R-phycoerythrin (PE)-conjugated antibodies (eBioscience Inc., San Diego, CA), each at 1∶25 dilution, and washed 3 times with PBS. Incubation with RmcB, anti-integrin α_v_β3/α_v_β5 antibodies, anti-syndecan 4, or 10E4 antibodies was followed by 1 hr incubation with Alexa 488-conjugated secondary antibody (1∶1000 dilution) and triple wash with PBS.

### Statistical Analysis

Microsoft Excel statistical software was used for a two-tailed Student's t-Test to determine statistical significance of the observed differences. The latter were considered significant at *P*<0.05.

## Results

### Establishment of Ameloblast-like Cells from an AI Patient

**Figure 1:**
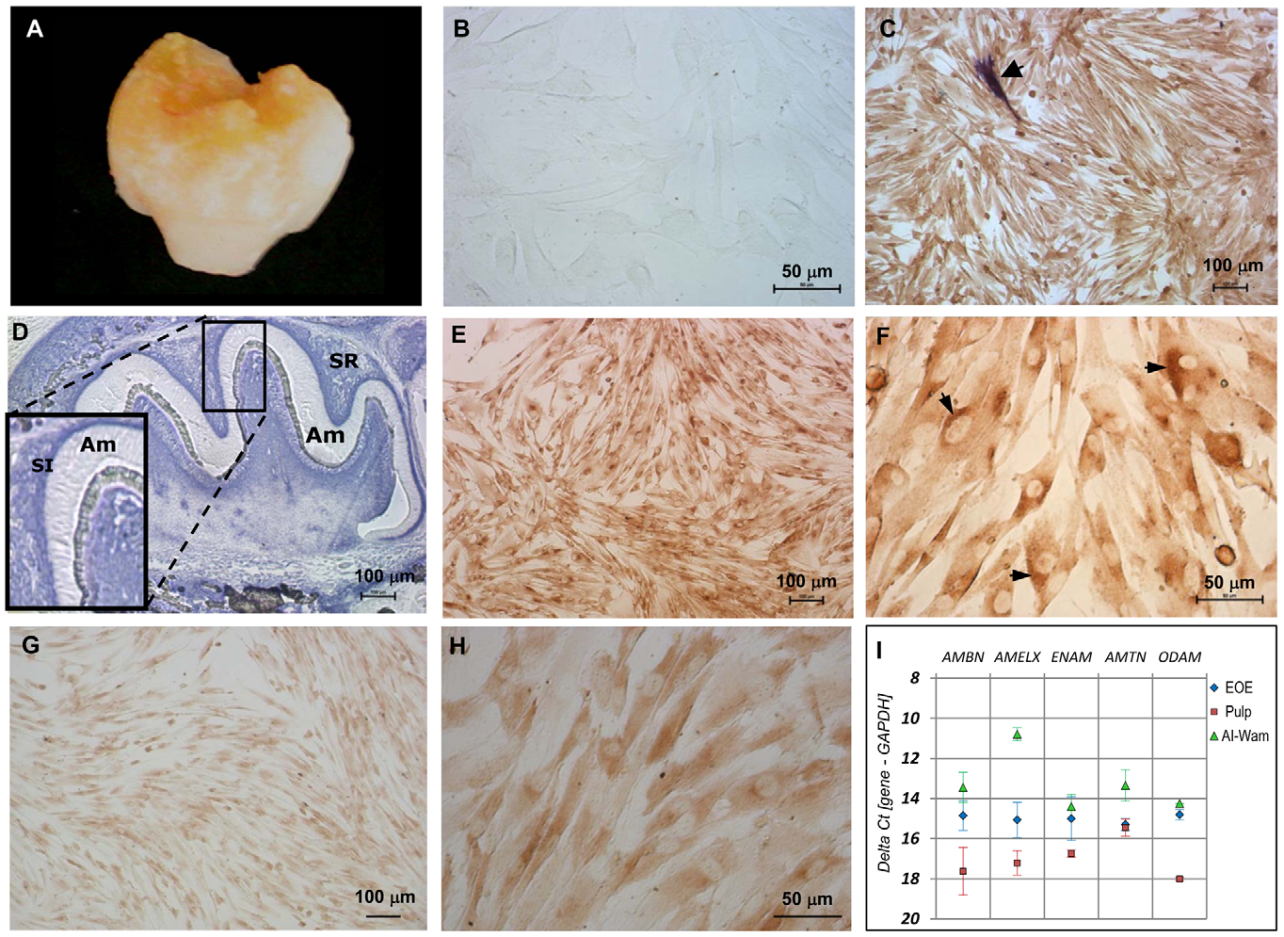
Characterization of the human AI-ameloblast cell population by immunohistochemistry, ALP in situ histochemistry and qRT-PCR analysis. **A.** Image of a tooth extracted from an AI patient that was used to establish an EOE primary cell culture. **B.** Phase contrast image of AI-WAm cell monolayer. **C.** AI-WAm cells stained for ALP activity followed by immunostaining for the epithelial marker cytokeratin 14; a single, highly ALP-positive cell is evident (arrow). **D.** Mouse molar stained for ALP activity showing ALP-negative secretory ameloblasts (Am) with highest activity (dark purple) in the stratum intermedium (SI) followed by the stellate reticulum (SR). Low (**E**) and high (**F**) magnification of AI-WAm cells positively stained for the major enamel protein Amel. Arrowheads on panel **F** indicate cells that appear polarized with unidirectional orientation of the Golgi apparatus. Low (**G**) and high (**H**) magnification of AI-WAm cells positively stained for the largest enamel protein Enam. **I.** Quantitative expression levels of the enamel matrix protein genes *AMBN*, *AMELX*, *ENAM*, *AMTN* and *ODAM* in AI-WAm cells relative to dental pulp and normal EOE cells as determined by RT-qPCR following normalization to a housekeeping gene *GAPDH* and presented as ΔΔCt values. Statistical analysis was carried out as described in Materials and Methods. Scale bars are: 50 µm (**B**, **F**, **H**), 100 µm (**C**, **D**, **E**, **G**).

Developing third molars (Fig. 1A) from a female hypoplastic AI patient were obtained after elective surgery and used to establish EOE explant cultures. A cell population termed AI-WAm (Fig. 1B) was isolated and confirmed to be of epithelial origin by cytokeratin 14 staining (Fig. 1C). This population contained less than 0.1% of ALP-positive larger cells, likely to represent stratum intermedium-like cells (Fig. 1C and D). The AI-WAm population was enriched for ameloblast-like cells, as shown by negative ALP staining (Fig. 1D), showing homogeneous Amel (Fig. 1E and F) and Enam (Fig. 1, G and H) expression at the protein level and mRNA level (Fig. 1I). Amel localization was intracellular with a polarized pattern (Fig. 1F, arrowheads). Furthermore, RT-qPCR analysis of these cells showed expression of mRNAs for all tested enamel proteins Ambn, Amel, Enam, Amtn and ODAM. As expected, all the enamel matrix protein genes, except *AMTN*, were expressed significantly higher in AI-WAm and normal EOE cell populations relative to the dental pulp cells (*P*<0.05), representing a control cell type of ectomesenchymal origin. While expression of the enamel protein genes was detectable in the dental pulp cells, their mRNA levels were extremely low (Fig. 1I).

### Susceptibility of AI-WAm Cells to Ad5 Transduction is Limited by hCAR Expression

**Figure 2:**
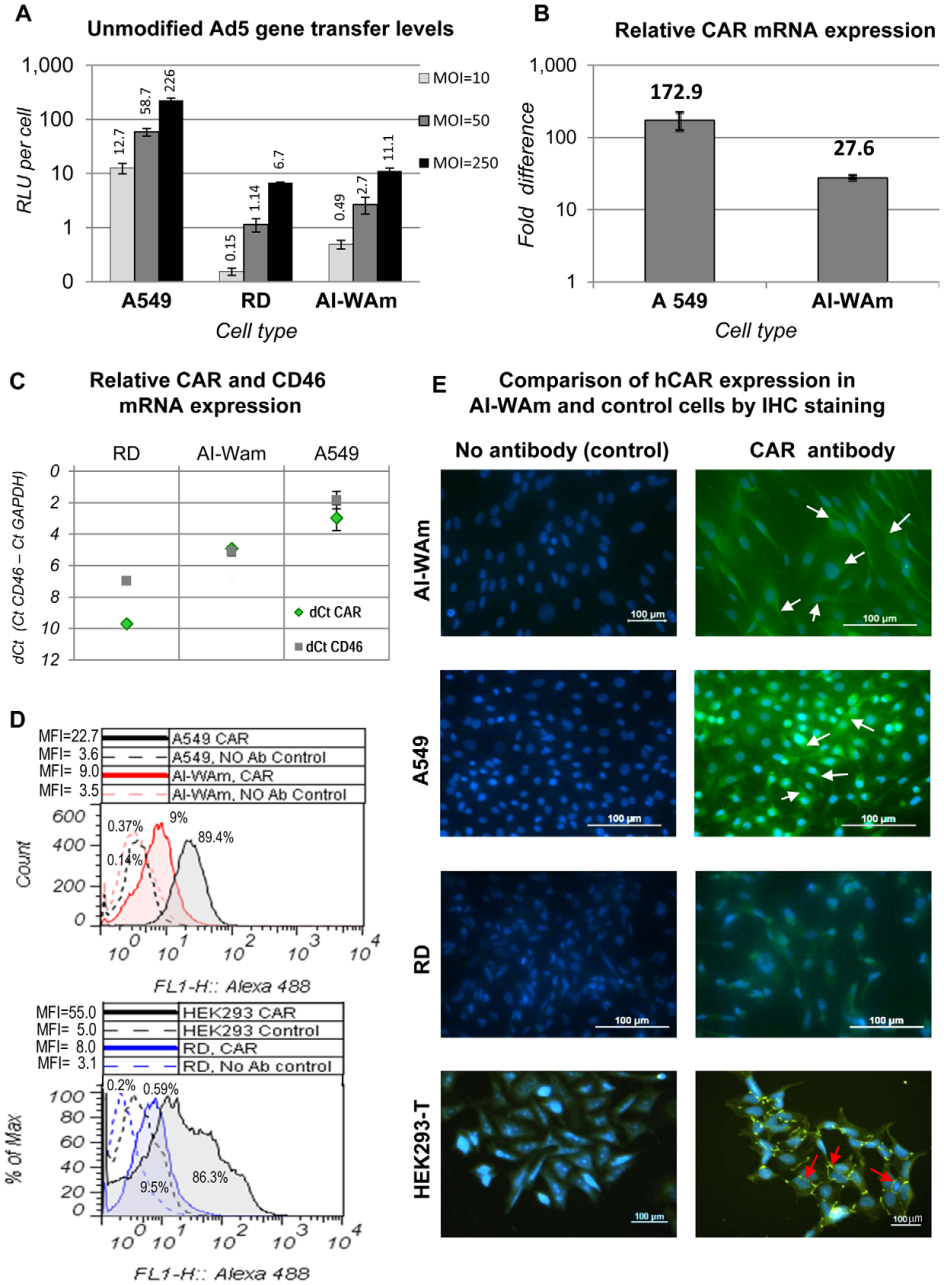
Ad5 gene transfer to AI-WAm cells is limited by deficiency in expression and/or cell surface localization of the Ad5 receptor CAR. **A.** Gene transfer efficiency of a human Ad5 vector expressing Luc reporter (Ad5 (L)) to an AI patient-derived ameloblast-like cells (AI-WAm) at different multiplicities of infection (MOI) (MOI = 10, 50 and 250 TCID_50_/cell) in comparison to CAR-positive A549 and CAR-negative RD cells by conventional Luc assay at 20 hours post infection. Results are presented in Relative Luc Units (RLU) per cell with mean values shown above each bar plus/minus standard deviation. All differences were statistically significant (*P*<0.05) **B.** Expression levels of hCAR mRNA in AI-WAm and A549 cells relative to that in RD cells as determined by qRT-PCR and presented as “fold difference”. All differences were statistically significant (P<0.05). *P_(A549/AI-WAm)_ = 0.027*. **C.** Quantitative analysis of hCAR and hCD46 mRNA expression levels in RD, AI-WAm and A549 cells as determined by qRT-PCR and normalized to the housekeeping gene *GAPDH*. The data are presented as ΔΔCt values. For AI-WAm *P_(CAR/CD46)_* = 0.44; for A549 *P_(CAR/CD46)_* = 0.127; *for RD P_(CAR/CD46)_* = 0.007; for CAR *P_(AI-WAm/A549)_ = 0.049*; *P_(RD/AI-WAm)_ = 0.033*; for CD46 *P_(RD/AI-WAm)_ = 0.0011*; *P_(AI-WAm/A549)_ = 0.0008*. *P*-values for all other differences were <0.05. **D.** Flow cytometry analysis of Ad5 receptor (CAR) expression in AI-WAm cell population in comparison with CAR-positive (A549) and CAR-negative (RD) control cells. Cells were incubated with primary anti-CAR (RmcB) monoclonal antibody (Ab) followed by labeling with Alexa 488-conjugated secondary antibodies. No primary Ab was used in negative control samples. The extent of shift in the fluorescent peak positions (color lines) relative to control peak(s) of unlabeled cells (black dotted lines) reflects the extent of cell labeling, corresponding to the receptor expression on each cell type and is expressed as Mean Fluorescence Intensity (MFI). Numbers above each peak correspond to percentage (%) of gated (M2) cells calculated using subjective gating. Fluorescence intensity (X-axis) is plotted as histograms on log scale (X-axis) using Flowjo 7.6.4 software (Tree Star Inc., Ashland OR). Y-axis depicts total events (cells) and expressed either as counts or % of maximal. *P_(AI-WAm/A549)_ = 0.0038*; *P_(AI-WAm/RD)_ = 0.39*; *P_(A549/RD)_ = 0.0018*; *P_(A549/HEK293T)_ = 0.018*; CAR *P_(AI-WAm/HEK293)_ = 0.0001*; *P_(HEK293/RD)_ = 0.001*; **E.** Comparison of hCAR expression in AI-WAm and control cells by IHC staining. CAR-specific (RmcB) primary antibody (same as used for FACS analysis, D) was used to stain AI-WAm cells. A549 and HEK293-T cells were used as positive and RD cells as negative controls for CAR expression. All cells were counter-stained for 5 min. with 300 nM DAPI to visualize nuclear DNA (blue). No primary antibody was used with control samples. Negative control (RD) cells show efficient nuclear staining but no discernible CAR staining (green), while A549 cells demonstrate a strong CAR-specific signal and AI-WAm cells display a moderate level of hCAR signal with diffuse pattern of cytosolic localization (white arrows) similar to that in A549 cells. In sharp contrast, CAR-overexpressing HEK293-T cells show a distinct localization of CAR protein in the cell membrane tight junctions (red arrows) with lesser cytosolic staining. Scale bars correspond to 100 µm.

Ad5-mediated gene transfer to AI-WAm cells was initially investigated by a conventional gene transfer assay using a capsid-unmodified Ad5 vector encoding firefly Luc as a reporter. While susceptibility of AI-WAm cells to Ad5 transduction was on average 23-fold lower than that of A549 cells, frequently used as a CAR-positive control [33], gene transfer to AI-ameloblasts was on average 3-fold more efficient than to “CAR-negative” RD cells [23] (Fig. 2A). In line with that, flow cytometry (FACS) analysis suggested a substantially lower level of hCAR protein in AI-WAm cells (MFI = 9 versus 3.5 of control) as compared to A549 (MFI = 22.7 versus 3.6 of control) or HEK293-T cells (MFI = 55 versus 5 of control) (Fig. 2D). Notably, hCAR expression in AI-WAm cells on mRNA level was only 6.3-fold lower than in A549 cells as revealed by RT-qPCR analysis (Fig. 2B and C). While immunohistochemistry (IHC) in AI-WAm cells demonstrated a weaker hCAR staining as compared to A549 cells, it was clearly positive relative to RD cells, frequently used as a “CAR-negative” control (Fig. 2E). Interestingly, in both A549 and AI-WAm cells hCAR showed mostly diffuse cytoplasmic localization in contrast to HEK293-T cells, used as another positive control for hCAR staining, where the receptor showed a distinct localization to the tight junctions of intercellular contacts (Fig. 2E arrows). In the aggregate, our data were consistent with lower expression of hCAR in AI-ameloblasts relative to A549 cells and especially to HEK293-T cells. The latter cells are known to be highly susceptible to transduction with unmodified Ad5 in agreement with almost a 15-fold higher expression of CAR in those cells on mRNA level (data not shown) and high level of CAR protein evidenced by nearly 10-times more labeled cells (86.3% versus 0.14% of control) as compared with AI-WAm cell population (Fig. 2D). Thus, limited susceptibility of AI-WAm cell population to infection with unmodified Ad5 vector was consistent with reduced expression and/or cell surface localization of the major Ad5 receptor hCAR in these cells.

### Capsid Modifications Improve Ad5 Gene Transfer to AI-WAm

**Figure 3:**
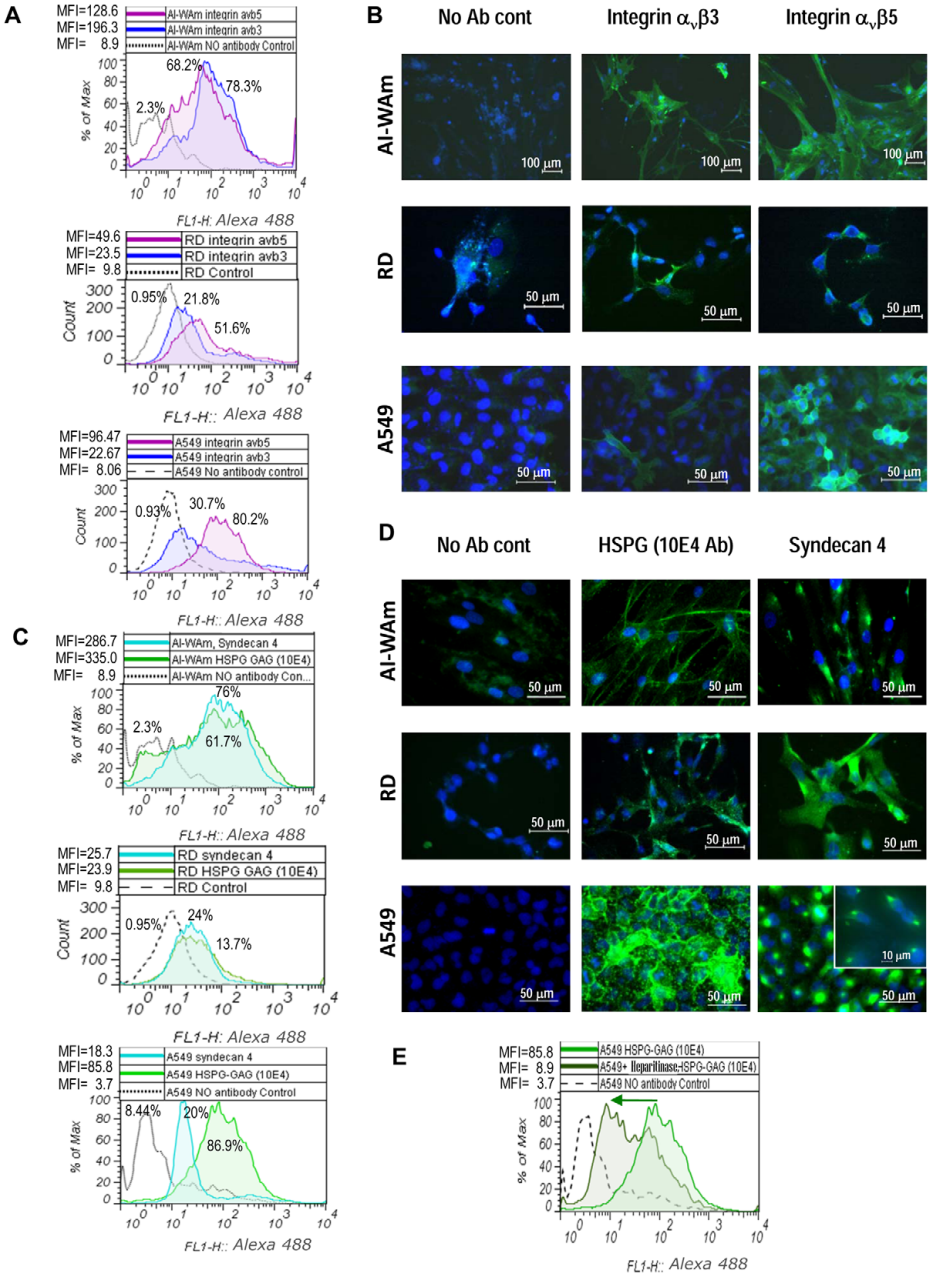
Expression analyses of HSPG and integrin molecules as alternate receptors for fiber-modified Ads on AI-WAm cells. Expression of α_v_β3 and α_v_β5 integrins (**A** and **B**) and heparin sulfate proteoglycans (**C–E**) in AI-WAm cell population and control cells was analyzed by flow cytometry (**A**, **C**, **E**) and IHC staining (**B** and **D**). Cells were incubated with primary anti-α_v_β3 or anti-α_v_β5 monoclonal antibodies for detection of corresponding integrin molecules or 10E4 antibody for detection of HSPG side chains (GAG) or anti-human syndecan 4 monoclonal antibody, followed by Alexa 488-conjugated secondary antibody. **A** and **C**, top charts: AI-WAm cells; middle charts: RD cells; bottom charts: A549 cells. For AI-WAm cells: *P*_(αvβ3/αvβ5)_ = *0.75*; *P*_(Synd4/HSPG)_ = *0.29*; For RD cells: *P*_(Synd4/HSPG)_ = *0.67*; *for* α_v_β3: *P_(RD/A549)_ = 0.38*; *for* α_v_β5: *P_(AI-WAm/A549)_ = 0.23*; *for syndecan 4: P*_(*RD*/A549)_ = *0.2*; *for* all other differences *P*<0.05; **E.** HSPG Ab (10E4) specificity control sample: A549 cells were treated with heparitinase (10 U/ml) for 1 hr at 37°C to remove GAG side chains. Green arrow shows shift of the fluorescence intensity peak resulting from reduction in cell labeling with 10E4 antibody (MFI decrease). Other details are as in **Fig. 2D**. **B** and **D**, scale bars correspond to: 100 µm in top image panels (integrins/AI-WAm, 10× objective), 10 µm (insert, 60× objective) and 50 µm (40× objective) in all other panels. Insert shows syndecan 4 staining image (60×) of A549 cells, clearly demonstrating a polarized intracellular localization of the protein.

**Figure 4:**
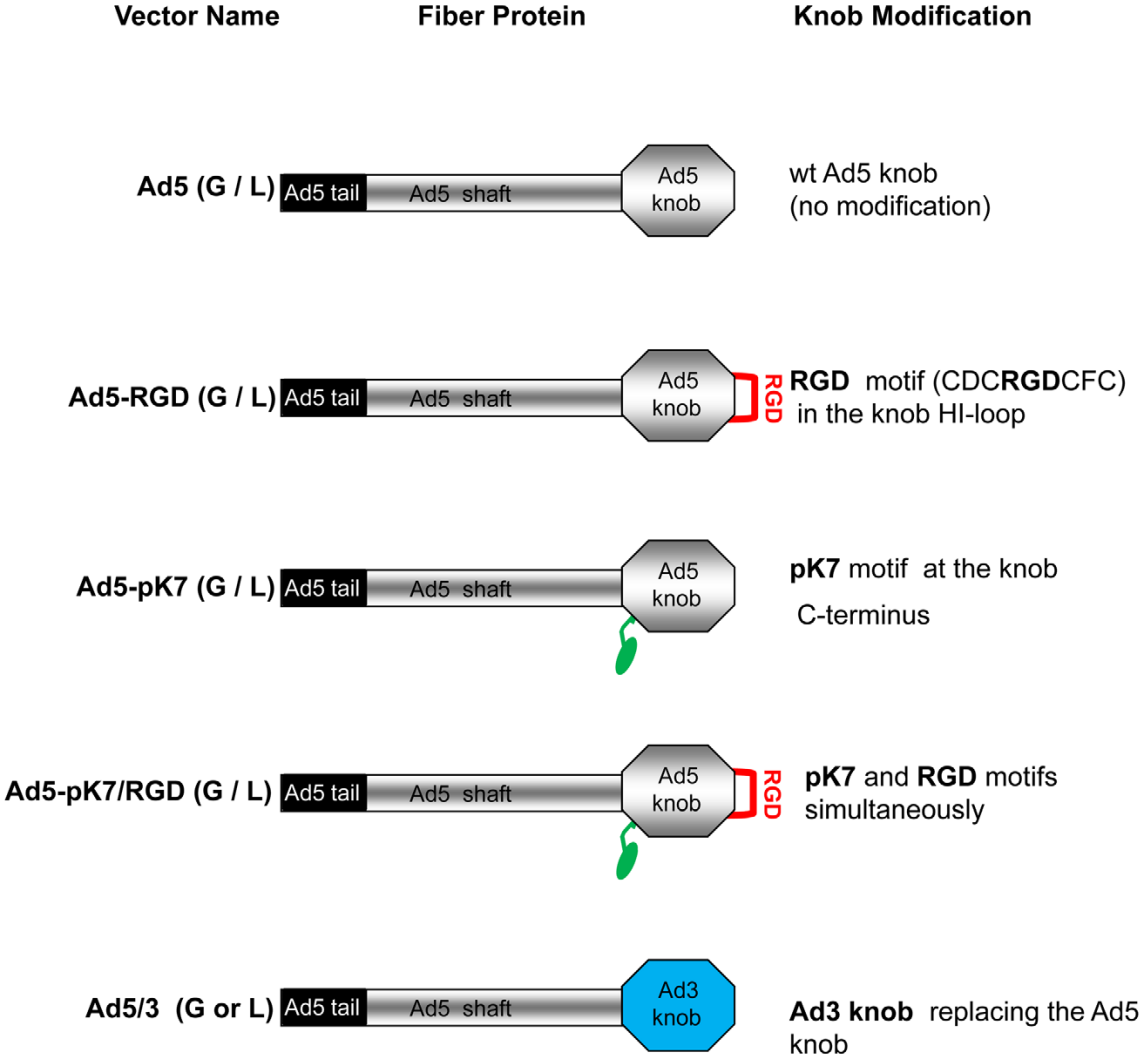
Schematic representation of the Ad5 fiber proteins carrying intact and modified C-terminal knob domains. Fiber modifications are indicated in the corresponding vector names: Ad5 (G/L) has unmodified fiber knob and possesses the native CAR tropism; Ad5-RGD contains a peptide ligand with an “RGD motif” in the HI loop (red loop) of the fiber knob; Ad5-pK7 contains a stretch of seven lysine residues (green oval) fused to the C-terminus of the Ad5 knob via a (GS)_5_ linker (green hook); Ad5-pK7/RGD incorporates both modifications in the corresponding locales of the same fiber molecule; Ad5/3 contains a chimera fiber with Ad5 fiber “knob” domain (gray) replaced with the Ad serotype 3 (Ad3) knob (blue), which retargets the vector to Ad3 receptor(s).

Since integrins α_v_β3 and α_v_β5 play a critical role in Ad5 internalization step [34], [35], we also examined AI-WAm cells for expression of these integrins by flow cytometry. The AI-WAm cells displayed a robust expression of α_v_β3 (78.3% of labeled cells, MFI = 196.3) as well as α_v_β5 integrins (68.2% of labeled cells, MFI = 128.6) (Fig. 3A). Expression of both integrins was also evident from IHC staining (Fig. 3B) that was consistent with the flow cytometry data. Taking into account higher expression of α_v_β3 and α_v_β5 integrins on AI-WAm cells, we reasoned that their transduction by Ad5 could be improved by genetic capsid modifications of the viral fiber protein with short integrin-binding cyclic peptide CDCRGDCFC (RGD-4C) bearing an arginine-glycine-aspartate (RGD) motif [36]–[38] incorporated in the HI-loop of the fiber knob domain (Fig. 4). This RGD-modification has been previously shown to greatly enhance Ad5 infectivity for α_v_ integrin-positive cells [39]–[41].

On the other hand, the abundance of negatively-charged heparan sulfate proteoglycans (HSPG) on the surface of epithelial cells known from the literature [42] suggested that a positively-charged heptalysine (pK7) peptide as an HSPG-targeting ligand [43] can be used for Ad5 fiber modification to enhance infectivity of Ad5 vectors in the ameloblast cell population.

Furthermore, we hypothesized that a double modification pK7/RGD (Fig. 4) could provide additional augmentation of Ad5 transduction in AI-WAm cells, based on the earlier finding that simultaneous incorporation of both pK7 and RGD ligands in the Ad5 fiber knob results in a synergistic effect in some CAR-deficient cells such as RD [23]. In order to confirm expression of HSPG on the surface of AI-WAm cells we carried out both flow cytometry and IHC staining using antibodies against syndecan 4, known to be up-regulated in AI-ameloblasts along with syndecan 2 and 3 [44], and 10E4 antibodies that recognize N-sulfated glucosamine residues of glycosaminoglycans (GAG) as HSPG epitope and commonly used to trace HSPGs [45]. As predicted, ameloblasts showed a robust expression of HSPGs and in particular, syndecan 4 (MFI of 335.0 and 286.7 versus 8.9 of control, respectively), which was also supported by an efficient IHC staining with the same antibodies (Fig. 3C and D).

It is noteworthy that syndecan 4-specific staining pattern was distinct from the pattern observed for total HSPG suggesting polarized localization of syndecan 4 versus a more uniform intracellular distribution of total HSPG. Syndecan 4 staining was also observed in CAR-negative RD cells and CAR-positive A549 cells showing even more conspicuous signal polarization in A549 cells (Fig. 3D, 3D insert). Expression of syndecan 4 in A549 cells was significantly (*P* = 0.0001) lower relative to total cellular HSPGs in contrast to AI-WAm cells (*P* = 0.286) and RD cells (*P* = 0.67) (Fig. 3C and D). Specificity of HSPG detection with 10E4 antibodies was evidenced by a profound drop in the cell fluorescence intensity upon pre-treatment of A549 cells with heparitinase (Fig. 3E, peak shift indicated by the green arrow), an enzyme specifically cleaving both N-acetylated and N-sulfated glucosaminido-glucuronic acid linkages of heparan sulfates and removing GAG side chains from cell surface HSPG molecules.

Finally, an Ad5/3 serotype chimera vector with fiber protein C-terminal knob domain genetically replaced with that of Ad3 (Fig. 4) was included in the panel of tested vectors because our experiments evidenced a substantial expression of tentative Ad3 co-receptors including HSPGs [46] and CD46 [47] on AI-WAm cells. Expression of CD46 in AI-ameloblasts was confirmed both on mRNA and protein levels using RT-qPCR (Fig. 2C), and flow cytometry (data not shown), respectively.

Taking into account that all the vectors with small peptide modifications of the fiber used in this study (except for Ad5/3) retain an intact CAR-binding site of the fiber protein and thus are potential CAR-binders, we utilized CAR-positive A549 cell line for dose normalization of all viral vectors using Luc reporter expression as a measure of Ad ability to bind to and internalize in those cells, i.e. as a surrogate “gene transfer equivalent”. Infectious doses of all vectors were empirically adjusted in A549 cells to provide equal or comparable Luc expression levels and then applied to CAR-negative or CAR-deficient RD and AI-WAm cells respectively to reveal relative effect of various fiber modifications on Ad5 transduction. In order to compare Ad5/3 with the panel of CAR-binding vectors we likewise adjusted its infectious dose to that of unmodified Ad5 and the other fiber-modified vectors by Luc expression levels in A549 cells. We reasoned that this strategy of Ad5/3 dose normalization would be valid provided comparable levels of Ad5 and Ad3 receptors in A549 cells. Comparison of CD46 versus CAR expression on mRNA level by RT-qPCR in various cells types suggested comparable gene expression levels for those genes in both A549 cells and AI-WAm cells but not in RD cells, which expressed a substantially higher level of CD46 mRNA relative to CAR mRNA (Fig. 2C). Although flow cytometry assay showed that A549 cells express high levels of both CAR (Fig. 2D) and CD46 (data not shown) proteins, precise quantitative assessment of their relative expression levels in this control cell line by FACS analysis was not feasible.

**Figure 5:**
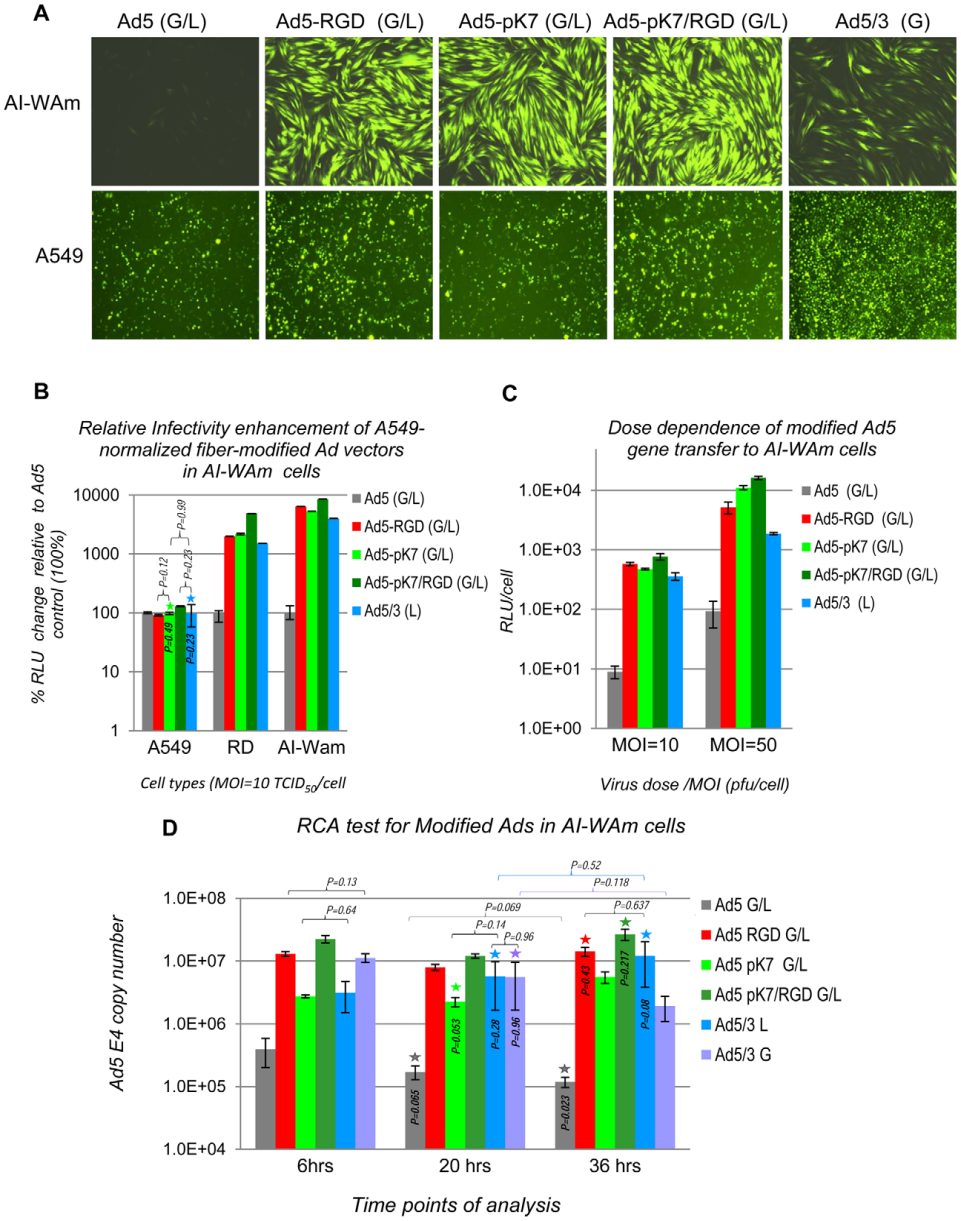
Augmentation of Ad5 gene transfer to AI-WAm cells by Ad5 vectors with various fiber modifications. **A.** Representative fluorescent microscopy images of cells infected with the array of recombinant Ad5 vectors with genetically-modified fibers shown on Fig. 4. Relative gene transfer efficiencies in the AI-WAm and A549 cells were assessed by fluorescent microscopy of GFP-expressing cells transduced with the array of modified vectors 20 hrs post infection; (G/L) indicates the presence of two reporter genes (GFP and Luc) each under control of its own CMV promoter; (G) denotes a single-reporter GFP cassette. Infection with Ad5 (G/L) was performed at an MOI of 50 TCID_50_/cell, while doses of other vectors were adjusted by Luc expression to match that of Ad5 (G/L) in A549 cells (see Materials and Methods and Results). A 50 TCID_50_/cell infection dose was used for Ad5/3 (G) vector for normalization in A549 cells as it could not be adjusted by Luc expression like the other (G/L) vectors. Fluorescent images were captured with 10× objective and exposure time of 2 sec. for A549 cells and 400 msec. for AI-WAm cells. Each vector infection was carried out in triplicates. Shown are images of representative samples; **B.** Luc assay of lysates prepared from A549, RD and AI-WAm cell samples (same as shown in A) following vector dose normalization in A549 cells using 10 µl of each lysate (100 µl) for bioluminescence analysis. The data are presented as percentage (%) of the Ad5 (G/L) gene transfer efficiency (total RLU) in each cell type taken as 100%; RLU - relative Luc units, (L) - a single firefly Luc reporter expression cassette. Other details are as in Materials and Methods. **C.** The original Luc assay data of the AI-WAm samples (shown in **A** and **B**) presented as RLU per cell. Two different MOIs of Ad5 (G/L) infection were used for dose normalization: 10 and 50 TCID_50_/cell to illustrate dose dependence of the infectivity enhancement effects. All other details are as in **B**. **D.** Test for replication competent adenovirus (RCA) contamination in modified Ad vector preparations by qPCR analysis of Ad genomic DNA. AI-WAm cells were infected with A549-normalized vectors at the doses corresponding to MOI = 50 of the Ad5 (G/L) (**C**) and harvested 6, 20 and 36 hrs post infection for total DNA isolation. Viral genomes were quantified by qPCR and presented as *E4* copy numbers normalized for total cellular DNA quantified by qPCR of the housekeeping (*GAPDH*) gene. No statistically significant increase in internalized genomic DNA evidences the lack of RCA-induced genomic DNA replication at 20 hours post infection (time point of Luc assay), suggesting that the reporter gene expression has not been affected for any vector. Color coding and other details are as in **B** and **C**. Stars above bars with the corresponding colors indicate changes relative to the 6 hr time point values with no statistical significance. *P* values (>0.05) are shown on the corresponding bars. Color brackets (with *P* values on the top) indicate value changes between 20 hr and 36 hr time points; black brackets (with *P* values on the top) show sample differences of no statistical significance (*P*>0.05) within the same time point. All other differences are statistically significant (P<0.05).

The use of two different reporters GFP and Luc, independently expressed by each vector, allowed for two independent readouts in the same experiment. Despite some subtle differences between the GFP (Fig. 5A) and the Luc (Fig. 5B) readouts, both reporters consistently revealed a dramatic augmentation of Ad5 infectivity in AI-WAm cells by all capsid-modified vectors relative to unmodified control with a superior effect of Ad5-pK7/RGD that was ranging from 85- to 405-fold depending on the multiplicity of infection (Fig. 5C). Notably, Ad5/3 showed the lowest augmentation of gene transfer in both AI-WAm and RD control cells relative to the other vectors, given its comparable transduction level in A549 cells following vector normalization (Fig. 5A and B).

Importantly, AI-WAm cells transduced with capsid-modified vectors, particularly Ad5-pK7/RGD, showed sustained expression of GFP in culture during 4 weeks (data not shown).

Validity of our approach of reporter expression-based vector dose normalization in A549 cells was supported by a direct quantification of the internalized Ad5 genomic DNA (*E4* copy number) by qPCR in a parallel experiment using the same viral doses as in the gene transfer experiments. As evidenced by Fig. 5D, the relative levels of the reporter gene expression seen in AI-WAm cells following infection with the fiber-modified vectors (Fig. 5B and C) recapitulate the rates of Ad5 genomic DNA transfer (estimated as *E4* copy number) to those cells upon vector transduction.

**Figure 6:**
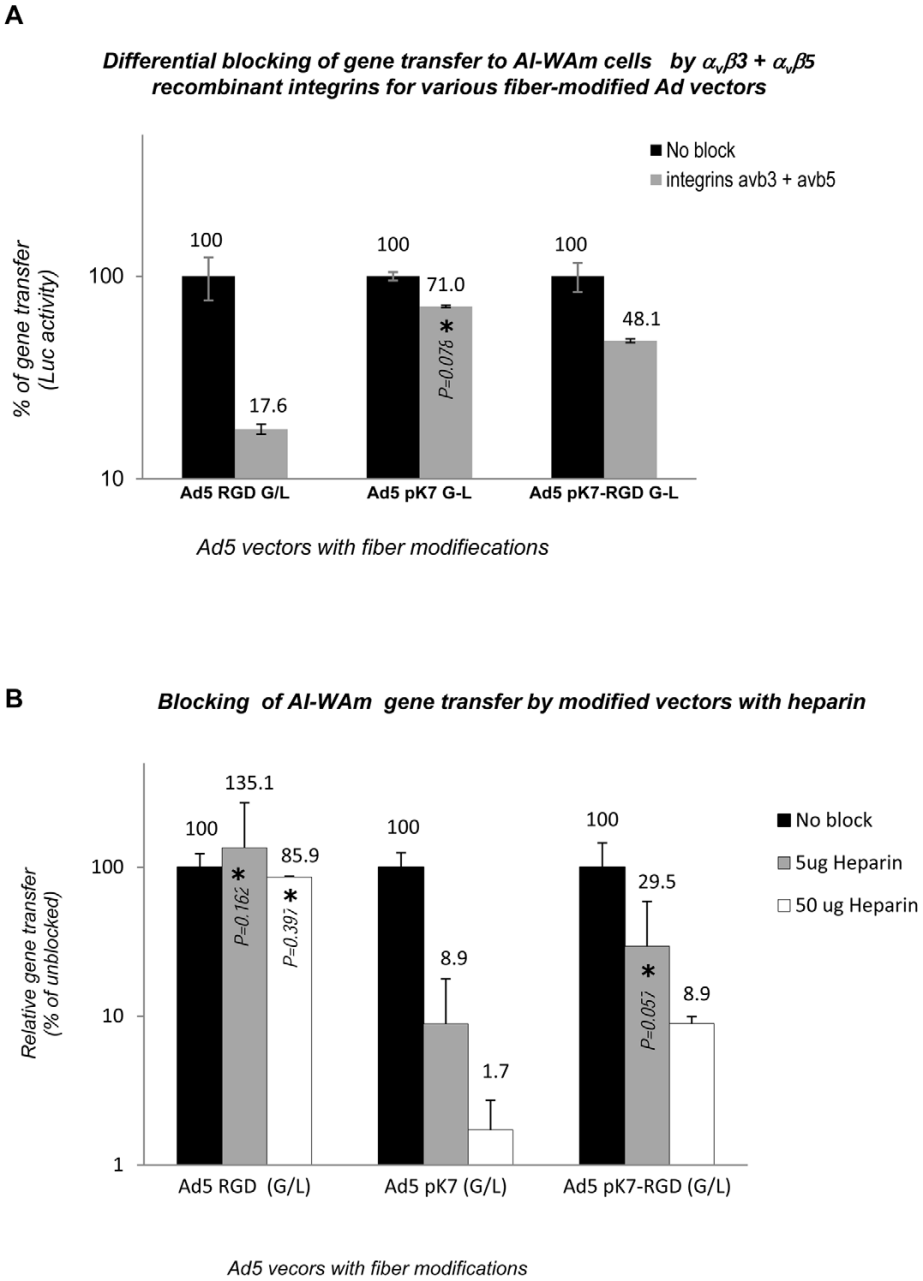
The infectivity enhancement effect of fiber-modified Ad5 vectors is mediated by α_v_β3/α_v_β5 integrins and/or HSPG molecules on AI-ameloblasts. **A.** Differential blocking of gene transfer to AI-WAm cells by integrins. Ad5 RGD shows the highest sensitivity to integrin blocking, while transduction with Ad5-pK7/RGD (G/L) is only partially inhibited. Ad5-pK7 (G/L) gene transfer shows no statistically significant inhibition by integrins. **B.** Blocking of AI-WAm gene transfer by modified vectors with heparin. Heparin shows a profound dose-dependent blocking effect on transduction with pK7-modified Ads, as opposed to RGD-modified vector. Gray bars (with % values on the top) show percentage of the residual gene transfer level (RLU) resulting from blocking relative to that of unblocked controls (100%) shown by black bar for each fiber-modified vector. All bars represent mean values with standard deviations. All differences were statistically significant except where indicated by asterisk and *P* values (*P*>0.05) on the data bars.

To verify that the mechanism for the observed infectivity enhancement is indeed mediated by binding of the modified Ads to α_v_β3/α_v_β5 integrins and/or HSPG molecules on the surface of AI-ameloblasts we carried out gene transfer blocking experiments using recombinant integrins or heparin as competitors for the respective targeted molecules on the surface of target cells. Pre-incubation of modified Ad vectors with 1∶1 mixture of purified α_v_β3 and α_v_β5 integrin proteins resulted in a profound blocking of AI-WAm gene transfer by Ad5-RGD (G/L) vector (17.6% of control) in contrast to Ad5-pK7 (G/L) (71% of control). Transduction by Ad5 RGD/pK7 (G/L) vector was also inhibited by integrins, but to a lesser extent (48.1% of control), supporting involvement of additional integrin-independent interactions (Fig. 6A). Conversely, when the viruses were pre-incubated with heparin as an HSPG competitor, the Ad5-pK7 G/L vector demonstrated a dramatic dose-dependent reduction in gene transfer, while neither Ad5-RGD (G/L) (Fig. 6B), nor Ad5/3 (L) (data not shown) vectors showed any statistically significant reduction in transduction of AI-WAm cells. The double-modified vector Ad5-pK7/RGD (G/L) showed a significantly lower sensitivity to gene transfer blocking with heparin, consistent with involvement of additional interaction of the modified vector with α_v_β3/α_v_β5 integrins via the RGD-4C ligand. Similar reciprocal blocking effects were observed for transduction of RD cells with the same panel of viruses (data not shown). Thus expression of syndecan 4 and possibly other HSPGs on the surface of AI-ameloblasts along with α_v_β3/α_v_β5 integrins allows circumventing CAR deficiency of those cells by using an integrin-binding RGD ligand in combination with a positively charged polylysine (pK7) incorporated in the Ad5 fiber knob domain.

## Discussion

We have established for the first time a human ameloblast-like cell population (AI-WAm) derived directly from an AI patient primary EOE tissue. The AI-WAm cells express *AMELX*, *ENAM*, *AMBN*, AMTN and *ODAM* genes at both mRNA and protein levels, which is consistent with the gene expression profile known for ameloblasts. Amel immunostaining showed cell polarization and was more robust than Enam staining, in line with the relative proportions of these proteins in the secretory ambeloblasts [13]. Since Amtn and ODAM are highly expressed in maturation- and postmaturation-stage ameloblasts [7], [8], [48], the AI-WAm cells, expressing relatively low levels of those gene transcripts (see Fig. 1I), are likely to represent the early maturation stage ameloblasts. Of note, both *AMELX* and *ENAM* genes have been excluded from a causative role in the disease pathogenesis for this AI patient (our laboratory unpublished data).

With this unique research tool in hand we next sought to evaluate the utility of Ad-based vectors for gene delivery to AI-ameloblasts as a potential strategy for gene replacement therapy of AI.

Efficiency of gene transfer by an Ad5 vector depends mainly upon the efficiency of the virus attachment to cellular receptors [49]–[53] and viral internalization [34], [35], [41]. Our initial experiments indicated that AI-WAm cells are about 23 times more resistant to Ad5 infection than A549 cells (see Fig. 2A), which was generally consistent with the lower levels of CAR expression observed in AI-ameloblasts on both mRNA (see Fig. 2B and C) and protein levels (see Fig. 2D and E). Although intracellular localization of hCAR is typically confined to tight junctions at the sites of intercellular contacts [54], the IHC staining for hCAR in AI-WAm revealed mostly diffuse cytoplasmic distribution of this protein (see Fig. 2E). Surprisingly, a similar hCAR staining pattern was observed also for hCAR-positive A549 cells, whereas in HEK293-T cells, used as another positive control in the same experiments, the protein showed a distinct localization to tight junctions (see Fig. 2E) in full agreement with the literature [54]. This difference between HEK293-T and A549 cells was most likely due to a substantially higher expression of hCAR in HEK293-T cells as suggested by a 15-fold higher expression of its mRNA in these cells as compared to A549 cells (data not shown). This agreed with the notion that A549 cells were more resistant to transduction with unmodified Ad5 as compared to HEK293-T or HEK293 cells (our unpublished observations). Other possible reasons for the observed difference in hCAR staining patterns between A549 and HEK293-T cells could be related to differences in physiological state (confluence, passage number) or growing conditions of those cells.

In addition to lower expression of hCAR in AI-WAm cells relative to A549 or HEK293-T control cells evidenced by our RT-qPCR and flow cytometry data, the IHC staining suggested paucity in cell surface-localization of the CAR protein, which could in part account for the observed resistance of AI-ameloblasts to Ad5 transduction.

To overcome the intrinsic CAR deficiency of AI-WAm cells we chose an approach of Ad5 infectivity enhancement using an array of Ad5 vectors with various genetic modifications of the capsid protein fiber that allow targeting of the vectors to alternate cell-surface molecules. We hypothesized that incorporation of a small cyclic peptide (RGD-4C) with affinity to cellular integrins (α_v_β3 and α_v_β5) [23], [36], [38], [39] or/and heptalysine peptide (pK7), targeting polysaccharide moieties of glycosylated cell surface proteins (HSPGs) [23], [43], could substantially increase Ad5 binding efficiency to cells of epithelial origin such as AI-WAm.

Expression of HSPG and α_v_β3/α_v_β5 integrins in AI-WAm and RD cells was confirmed by IHC staining and flow cytometry analysis (Fig. 3). The flow cytometry data were generally consistent with IHC staining of cells for HSPGs and integrins. The staining patterns for both integrins (α_v_β3 and α_v_β5) were similar in all cell types with both diffuse cytosolic distribution and cell membrane-localized signals (Fig. 3B). In contrast, staining patterns for total HSPGs based on detection of GAG moieties was very distinct from that for syndecan 4, which showed a highly polarized localization throughout all analyzed cell types (Fig. 3D), particularly conspicuous in A549 cells (Fig. 3F). This observation was consistent with specific intracellular localization of syndecan 4 to complex cytoskeletal adhesion sites, i.e., focal adhesions [55]. Of note, expression of syndecan 4 in ameloblasts was higher than in A549 cells relative to total HSPG in those cells (Fig. 3C and D).

Adequate comparison of Ad vectors with various tropisms in the context of the established dental cell population was an important, but challenging task due to the problem of proper dose normalization of vectors carrying different tropism modifications. Gene transfer assay with reporter gene expression as readout is typically employed to compare transduction efficiencies of viral vectors using either “physical” or “infectious” titers for vector dose normalization. Vector dose normalization for gene transfer experiments has been a subject of controversy in the field because the level of transgene expression in infected cells may not be reflective of the number of viral particles (VP) used for transduction or infectious (pfu/TCID_50_) titer i.e. viral ability to form infectious progeny and produce plaques on infected cell monolayer. Moreover, Ad infectious titers are typically determined in helper (HEK293 or 911) cell lines regardless of tropism of analyzed vectors, which potentially leads to under- or overestimation of vector infectivity in other cell types with different repertoire of cell surface receptors.

To minimize potential errors in vector dose normalization due to the aforementioned factors we sought to employ a different approach based on empirical adjustment of reporter gene (Luc) expression levels for each virus in CAR-positive A549 cells as an equivalent of Ad5 infectious dose or “transgene expression dose”. We reasoned that the efficiency of vector-encoded transgene expression in a cell line with high levels of the native Ad5 receptor (CAR) represents an “equivalent” of gene transfer (cell binding and internalization) capability of fiber-modified Ad5 vectors that retain their natural ability to bind CAR (non CAR-ablated). Our rationale was based on the assumption that in CAR-expressing cells transduction by infectivity-enhanced vectors would occur predominantly via the native cognate receptor (CAR) pathway with relatively lesser contribution of other receptors provided small ligand modifications of the fiber do not significantly compromise CAR tropism of the vectors. On the contrary, in CAR-deficient or CAR-negative cells lines the cell binding mechanisms mediated by Ad5 fiber modifications would become predominant and determine transduction efficiency of the modified vectors.

The validity of our approach for viral dose normalization in A549 cells has been supported by the results of direct quantification of internalized genomic DNA (*E4* copy number) for each modified virus in A549 (data not shown) and AI-WAm cells (see Fig. 5D). This analysis showed direct correlation between the Luc expression readouts following vector dose normalization and the actual Ad5 gene transfer levels assessed by intracellular quantification of Ad5 genomic DNA using the *E4* region-specific qPCR (see Fig. 5D).

This study demonstrated that each tested fiber modification drastically enhanced Ad5 infectivity in CAR-deficient cells such as RD and AI-ameloblasts (see Fig. 5). The observed synergistic effect of the two small ligand modifications in the Ad5-pK7/RGD (G/L) vector was consistent with the original report [23] using RD as CAR-negative cells for evaluation of pK7 and/or RGD modification and suggested that pK7/RGD double modification represents an optimal fiber modification also for gene transfer to AI-ameloblasts at therapeutically relevant doses.

Our reciprocal blocking experiments with heparin or a mixture of recombinant α_v_β3 and α_v_β5 integrins demonstrated that the mechanism for the observed infectivity enhancement of Ad5-RGD (G/L) and Ad5-pK7 (G/L) vectors indeed involves their binding to α_v_β3/α_v_β5 integrins and HSPGs, respectively. Our experiments also suggest transduction through both types of molecules for double-modified Ad5-pK7/RGD (G/L) virus (Fig. 6). The same mechanism apparently mediated augmentation of transduction of control RD cells with negligible expression of CAR but substantial expression of HSPGs, including syndecan 4, since transduction of these cells was blocked by heparin with similar efficiency (data not shown). Although our blocking data generally agreed with the earlier study [23], we observed a substantially more robust inhibition of gene transfer by both integrins and heparin (Fig. 6). Despite higher doses of heparin used in our experiments, which could account for stronger blocking effects (58-fold and 11.2-fold for Ad5-pK7 and Ad5-pK7/RGD, respectively, versus 3.3-fold and 1.5-fold inhibition reported for the respective vectors in RD cells previously), it had no inhibitory effect on Ad5-RGD vector transduction in AI-WAm cells (Fig. 6B), in contrast to the slight effect in RD cells reported previously [23]. The reason for this discrepancy is unclear and could reflect subtle differences in the cell-binding mechanism of the HSPG-targeted Ads in AI-WAm versus RD cells.

Integrins α_v_β3 and α_v_β5 have been implicated in internalization of the group C adenoviruses (Ad2/Ad5), which involves their interaction with an RGD motif of the Ad capsid's penton base [34]–[38], [41] following the fiber knob-mediated step of the viral attachment to CAR [20], [33], [49]–[53]. The affinity of RGD interaction with α_v_β3 and α_v_β5 integrins is relatively lower than that of the fiber-CAR interaction, although it is essential for triggering formation of endosomes as well as for Ad5 endosome escape. The latter step requires α_v_β5 (β5 cytolasmic tail) but not α_v_β3 for endosome membrane permeabilization critical for Ad5 entering the cytoplasm. Of note, differential role of α_v_β3 and α_v_β5 has been suggested more recently for transduction of RGD-modified Ad (Ad5-RGD), implicating primarily α_v_β3 in binding to linear RGD peptide of the penton base as well as to the cyclic peptide RGD-4C in the fiber knob [56]. In this regard, robust expression of α_v_β3 on AI-WAm is consistent with strong augmentation of the Ad5-RGD (G/L) vector transduction of those cells observed in this study, whereas higher overall expression levels of HSPGs and integrins in AI-ameloblasts relative to RD cells was consistent with more efficient transduction of AI-WAm cell population with the RGD and/or pK7-modified viruses.

Because both replicative and replication-deficient Ad5/3 vectors carrying a chimera fiber modification demonstrated superior infectivity enhancement in different types of cancer cells both *in vitro* and *in vivo* [57]–[62], it was of interest to evaluate efficacy of this modification for transduction of non-cancer epithelial cells such as AI-WAm relative to the small modification ligands. However, Ad5/3 dose normalization to the panel of CAR-binding vectors presented a problem due to ablated CAR tropism of the virus resulting from the replacement of the entire Ad5 fiber knob domain with that of the serotype 3 Ad (Ad3) known to target a different set of cellular receptors [24], [63]. Several groups have identified the membrane cofactor CD46 as an attachment receptor for human Ad group B serotypes, including Ad11 [64], Ad35 [65] and Ad3 [47]. Furthermore, high-throughput receptor screening approach identified HSPGs as low-affinity Ad3 co-receptor interacting with its fiber knob domain [46]. It has thus been suggested that more than one receptor exists for species B Ads [46], [47], [64]–[67]. It remains controversial whether CD46 functions as attachment receptor for all species B serotypes. Most recently, the major Ad3 receptor was identified with desmoglein 2 (DSG2) [68], making the relevance of CD46 to the mechanism of Ad3 transduction highly questionable.

Our strategy of Ad5/3 infectious dose normalization to those of the fiber-modified CAR-binding Ads was originally based on the assumption that Ad5/3 and Ad5 possess similar abilities to transduce A549 cells resulting from comparable levels of CAR and Ad3 receptor(s). Due to the fact that this study was carried out prior to the discovery of DSG2 as a primary Ad3 receptor, in this study we analyzed expression of CD46 as a tentative Ad3 receptor [51] on A549 cells and found comparable expression of this molecule to CAR at least on mRNA level (Fig. 2C). We also found high levels of CD46 and CAR proteins in A549 cells (Fig. 2D and E), in line with earlier reports [28], but were unable to determine the relative ratio of those molecules quantitatively using flow cytometry approach since anti-CAR and anti-CD46 antibodies could have different affinities and cell labeling with different antibodies might not reflect the actual level of each receptor. Furthermore, after DSG2 was identified as the major Ad3 receptor, the analysis of CD46 expression in A549 cells became no longer relevant to the assessment of the bona fide Ad3 receptor levels and justification of our Ad5/3 normalization approach. In this regard, to validate our strategy for Ad5/3 normalization in A549 cells we employed a novel quantitative approach developed in a separate study to determining relative efficiency of A549 cell transduction by Ad5 and Ad5/3 vectors. Briefly, we used Ad5 and Ad5/3 viruses with EGFP-labeled capsids [68] as fluorescent tags in flow cytometry assay to probe A549 cells for the corresponding cognate receptors under conditions of receptor saturation and viral internalization block (4°C). Considering that the viruses had comparable infectious titers and capsid labeling efficiencies, the difference in Mean Fluorescent Intensities (MFI) of the cells bound to viral particles of each type (peak positions) relative to MFI of unlabeled cells (background) was reflective of the difference in A549 cell binding capacity of each vector (Ad5 or Ad5/3) under receptor saturation conditions. The above-mentioned approach revealed that A549 cells were capable of binding somewhat larger number of Ad5/3 particles than Ad5 particles (MFI = 109.4 versus MFI = 38.2, with 97% and 94% labeled cells, respectively). In light of these findings transduction efficiency of Ad5/3 vector (normalized to other vectors in A549 cells) observed for AI-WAm cells is likely to be underestimated and could potentially be higher than observed in our experiments (see Fig. 5). This could account for the relatively lower gene transfer augmentation demonstrated by the Ad5/3 vectors (L or G) in AI-ameloblasts as compared to the other fiber-modified vectors (Fig. 5B and C). Although expression of DSG2 in pre-ameloblasts was evidenced by gene expression arrays (our unpublished observations), expression of this protein in AI-WAm cell population may be down-regulated relative to HSPG and integrins, which could also account for the relatively lower augmentation of their transduction by the chimera Ad5/3 vectors.

This initial study thus defines an optimal gene delivery strategy to ameloblast-like cell population derived from a human patient with AI. Extrapolating *in vitro* results to clinical situation is a common challenge of the gene therapy field, although some general principles that have been derived from studies in cell lines have been supported by observations from clinical trials [69]. Ad-mediated gene transfer is dose-dependent, but increasing Ad doses inevitably leads to enhanced host immune response to Ad vectors and systemic toxicities. Therefore, the superior efficiency of gene transfer to AI-ameloblast cell population demonstrated by some capsid-modified vectors in this study along with the observed longevity of transgene expression (up to 4 weeks) in the target cells offers a potential utility of fiber-modified Ad5 vectors for local administration in dental tissues.

Several reasons warrant hope for Ad gene therapy applications in dentistry. Often, the typically limited duration of Ad5-delivered transgene expression *in vivo* [70]–[72] is a crucial obstacle, but in developing teeth relatively short-term changes (weeks or months) in matrix protein expression can determine the properties of the mineralized tissues. Accordingly, a localized Ad-mediated gene delivery strategy, rescuing essential components of the developing tooth in a temporal fashion, could restore the complex choreography of mineralized matrix formation during a critical time window. Moreover, localized administration of an Ad5 vector allows minimization of its clearance by pre-existing anti-Ad 5 antibodies [71], [72]. Successful induction of bone formation by bone morphogenic protein (BMP), delivered via an Ad gene therapy vector by its localized microsurgical infusion [73], supports feasibility of Ad gene therapy applications also for morphogenesis of dental tissues during permanent tooth formation. These perspectives warrant further evaluation of the Ad5-pK7/RGD as a potential AI-gene therapy vector in a suitable animal model of amelogenesis as a logical next step in the above research strategy.
